# Optimizing Usability of Digital Health Interventions for Nondigitally Native Adults: A Framework-Based Approach to Develop Just-in-Time Adaptive Interventions (JITAIs) and Other Adaptations

**DOI:** 10.2196/87365

**Published:** 2026-08-11

**Authors:** Abby L Cheng, Christine Y Gou, Adriana Martin, Sarah M Hartz, Joanna Abraham

**Affiliations:** 1 Division of Musculoskeletal Physical Medicine and Rehabilitation, Department of Orthopaedic Surgery, Washington University in St. Louis, Campus Box MSC 8233-0004-05, 660 S Euclid Ave, St. Louis, MO, 63110, United States, 1 314-747-8489; 2 Department of Psychiatry, Washington University in St. Louis, St. Louis, MO, United States; 3 Department of Anesthesiology and Institute for Informatics, Washington University in St. Louis, St. Louis, MO, United States

**Keywords:** mental health, depression, anxiety, chronic pain, mHealth, usability, user experience, satisfaction, digital health, digital mental health intervention, just-in-time adaptive intervention, middle-age, older adult

## Abstract

**Background:**

Health-related technology use among nondigitally native adults is becoming widespread. Nevertheless, digital health interventions are typically not designed with the unique usability needs and preferences of this population in mind. Furthermore, most of these middle-aged and older adults are managing multiple medical conditions, which can also impact their preferences related to digital health interventions. A prime opportunity to address multimorbidity in nondigital natives using a digital health intervention is the intersection between mental health and chronic pain.

**Objective:**

The goal of this study was to use established frameworks and end-user feedback to identify actionable, usability-related features that can be incorporated into existing digital health interventions and that are preferred among nondigitally native adults. We aimed to identify general usability adaptations, as well as just-in-time adaptive interventions (JITAIs).

**Methods:**

We conducted a qualitative usability study of a convenience sample using a hybrid inductive-deductive content analysis to evaluate an existing mental health app (Wysa for Chronic Pain). Participants were 45 years or older; reported at least moderate symptoms of depression or anxiety (Patient Health Questionnaire -9 ≥10 or Generalized Anxiety Disorder -7 score ≥10); and endorsed having pain at least most days in the past 3 months. The Framework for Reporting Adaptations and Modifications to Evidence-based Implementation Strategies was used to identify potential usability-related adaptations for the target population. Development of usability-related JITAIs was guided by Nahum-Shani’s pragmatic framework for JITAI development and the Behavioral Integration Technology model.

**Results:**

Forty-two participants completed usability testing (mean age 57, SD 8 years; women n=32, 76%). Participants identified numerous opportunities to optimize their user experience, primarily by ensuring the app clearly describes how all its features are intended to be used and by minimizing navigation burden. Specifically, participants requested clear, step-by-step orientation and navigation instructions, rather than a brief onboarding experience that relies on user-led exploration and familiarity with conventional app symbols. Participants also recommended a customized experience based on their unique usage patterns. The most common JITAI opportunity identified was to strategically reduce user notifications in order to reduce the risk of notification fatigue and subsequent complete disengagement with the app.

**Conclusions:**

Identifying actionable opportunities to improve usability and prompt engagement with “the right tool at the right time for the right person” holds promise to improve the effectiveness of digital health interventions across the age span. The usability-related refinement opportunities identified in this study are generalizable to other digital health interventions that are relevant to older users who are likely to be managing multimorbidity, are not digital natives, and are more likely than younger users to have physical or cognitive challenges. The integrated framework-based process described in this article can also serve as a model for optimizing other existing digital health interventions.

## Introduction

Most digital health interventions are not equally “accessible” to everyone [1]. Specifically, they are typically not designed with the usability needs and preferences of nondigital natives (born before 1980) in mind [2,3]. Technology use is rapidly growing across the age span [4-6], but compared to younger users who grew up “digitally native” with widespread access to technology and for whom many interventions were initially designed, middle-aged and older adults tend to have different habits and characteristics. For instance, they tend to check their mobile devices less frequently, and most do not instinctively search for a technological solution to a problem. In addition, this older population often experiences age-related changes in visual acuity and eye tracking patterns, and some have age-related dexterity or cognitive deficits [4,7-10]. Furthermore, the prevalence of multimorbidity increases with age [11]. As a result, nondigitally native adults tend to prefer different screen layouts than younger technology users; they may benefit from more “push” notifications, and they are more often interested in interventions that address multimorbidity [4,5].

A growing body of evidence is available to guide developers in understanding the usability preferences of nondigitally native adults [4,5,12], but a gap still exists in translating these general preferences into real interface changes that are implemented within existing digital health interventions. Furthermore, just-in-time adaptive interventions (JITAIs) are a relatively new and persistently underused method that aim to deliver the “right intervention at the right time to the right person,” and they have the potential to reduce the need for users to initiate interaction with a digital health intervention or to navigate through the intervention independently [13]. We hypothesize that JITAIs may be particularly impactful to improve usability for nondigitally native adults, but they are not yet widely applied to publicly available digital health interventions [14]. Guiding frameworks have also been developed in recent years that can facilitate optimal reach and effectiveness of digital health interventions, but they, too, have not been routinely used in intervention development [15]. Applying these frameworks and JITAI capabilities to adapt existing interventions for key target populations could rapidly improve the accessibility and subsequent effectiveness of publicly available digital health interventions.

A prime opportunity to address multimorbidity in nondigitally native adults using a digital health intervention is at the intersection between mental health and chronic pain [16-21]. Digital mental health interventions are now widely available to address depression and anxiety symptoms [22-27], in large part because they can be effective and improve access to mental health support for people who otherwise may be limited by barriers such as stigma, cost, or geography [27-32]. Nevertheless, chronic pain commonly coexists with symptoms of depression and anxiety, and it reduces the effectiveness of mental health treatments unless pain is simultaneously addressed [20,33-38]. Furthermore, the prevalence of chronic pain increases with age [20,35].

The goal of this study was to use established frameworks and end-user feedback to identify usability preferences among nondigitally native adults for digital health interventions. We specifically focused on nondigitally native adults with chronic pain and coexisting symptoms of depression or anxiety, and we aimed to obtain feedback on participants’ preferred general usability adaptations, as well as usability-related JITAIs. Our motivating scientific premise is that incorporating usability-related adaptations will improve overall engagement and subsequent effectiveness of digital health interventions, and evaluation of this potential relationship will be assessed in future work.

## Methods

### Study Design

This was a formative, qualitative usability study in which participants from the target population of interest completed usability testing of an existing mental health app. Usability testing occurred in 2024, and data analysis occurred in 2024 and 2025. Of note, additional feedback from study participants regarding contextual determinants of engagement with mental health apps was reported separately [12].

This study was performed via an academic-industry partnership. Academic investigators had full independence in designing the study, recruiting participants, conducting the usability testing, performing data analysis, and reporting study results. The industry partner provided access to the mental health app under investigation and created mock-ups of refined app screens and workflows for the iterative usability testing process. The academic investigators shared the study findings with the industry partner so that the industry partner could refine its mental health app accordingly.

### Ethical Considerations

Ethical approval was obtained by the Washington University Institutional Review Board via a waiver of written informed consent prior to participant recruitment (Institutional Review Board ID 202311024). Participant data were maintained in secure databases and were deidentified before sharing outside the institutional review board–approved study team. Participants received a US $30 gift card for participation.

### Participants or Users

Participants were at least 45 years old, reported at least moderate depression or anxiety symptoms (Patient Health Questionnaire-9 ≥ 10, Generalized Anxiety Disorder-7 score ≥ 10, or both), and endorsed chronic pain (defined as pain on most days or every day in the past three months) [20,39-41].

Exclusion criteria included endorsement of an active mental health crisis that warranted escalation of care (eg, active suicidal ideation and psychosis), cognitive impairment that would interfere with communication with the researcher or meaningful engagement with the mental health app (assessed informally by the research coordinator asking the potential participant to restate the study purpose, activities, and risks, benefits, and alternatives of study participation during the informed consent process), and residing outside the United States.

### Recruitment

Participants were a self-selected convenience sample recruited from multiple sources including a chronic musculoskeletal pain clinic at a US academic medical center, email, and social media advertisement via the medical center’s volunteer research participant registry, and targeted advertisement in a free online health community (The Mighty). Eligibility screening was performed using a self-reported questionnaire hosted by REDCap (Research Electronic Database Capture; Vanderbilt University), followed by verification by the research coordinator [42,43]. Recruitment was closed when usability testing by additional participants no longer yielded new themes within the convenience sample during the iterative analysis process [44].

### Intervention Assessed in Usability Testing

The mental health app assessed in usability testing, Wysa for Chronic Pain, aims to address mental health and chronic pain through a multifaceted approach by facilitating behavioral activation, pain acceptance, and improved sleep quality [45-48]. It guides users through evidence-based cognitive behavioral therapy, mindfulness-based interventions, and sleep support. The app has used a rule-based conversational agent with natural language understanding to deliver therapeutic content since 2016. In 2024, it augmented this approach with the addition of large language models at specific nodal points. Large language model–based capabilities were integrated into check-in conversations to improve the chatbot’s ability to understand context-specific content over multiple interactions and respond in a more personalized manner to the user. However, risk-based and crisis conversations have remained completely rule-based and written by a clinician. Although the parent Wysa company offers multiple commercially available products, Wysa for Chronic Pain is not currently commercially available.

**Figure 1.**
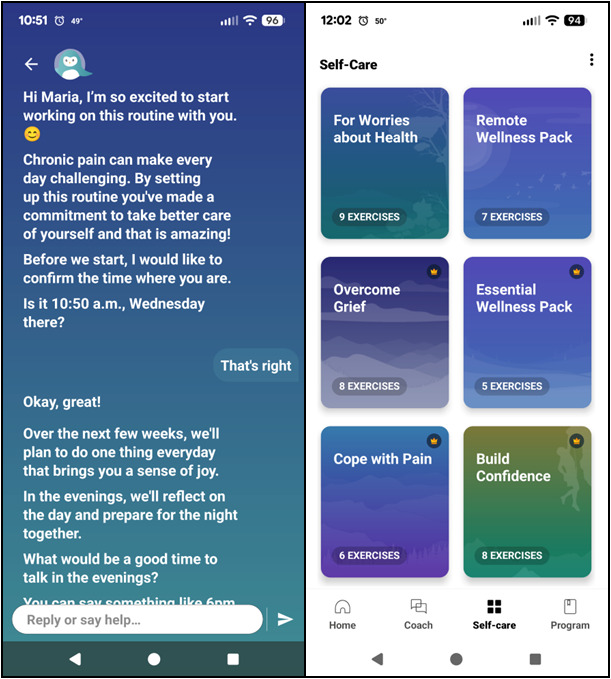
Screenshots of Wysa for Chronic Pain.

The therapeutic tools within Wysa for Chronic Pain are designed to be delivered via a combination of a structured curriculum and optional user-led exploration. By default, the app sends push notifications to users twice daily. The morning notification prompts an activity scheduling and planning task, and the evening notification prompts an activity tracking and mood reflection task and then offers a sleep meditation (Figure 1). Additional therapeutic content such as cognitive restructuring, thought recording, journaling, anxiety management, etc is delivered in a weekly cadence over 8 weeks. Each week, users receive a push notification that directs them to a report that details their app engagement thus far. Accessing the report also launches a congratulatory message for their engagement and unlocks access to new therapeutic tools as a reward for their engagement. Users can also independently navigate to therapeutic tools at any time, which are organized into “tool packs.” As a safety feature, the app also includes an “SOS” safety plan feature, which lists crisis hotlines and allows users to enter and store self-reported sources of hope and “safe people or places” to reference in moments of crisis.

### Usability Testing Process

Participants answered sociodemographic questions via an electronic REDCap questionnaire for descriptive purposes. Usability tests were conducted and recorded using audio and visual conferencing via a secure Zoom (Zoom Video Communications, Inc.) platform, each lasting approximately 45‐60 minutes. After participants downloaded Wysa for Chronic Pain to their mobile device, they were asked to complete the app’s onboarding process and complete several tasks within the app, all while performing “think aloud” testing, during which they described their thoughts and experiences in real-time (Multimedia Appendix 1: interview guide). During this process, the facilitator also took note of participants’ hesitations and inaccuracies in navigating the app, and the facilitator requested feedback on potential JITAIs that could be incorporated into the app. JITAI options and their details were iteratively refined throughout the usability testing process via a rapid iterative testing process. Based on feedback from the first 21 participants, mock-ups of revised app screens and workflows were created and shared with the next 13 participants using Figma (Dev Mode) and Twine (Chris Klimas) software. Additional minor refinements were made to the mock-ups for review by the final 8 participants. However, the evaluation tasks and the app’s core functionalities remained consistent for all participants.

### Analysis Frameworks

**Figure 2.**
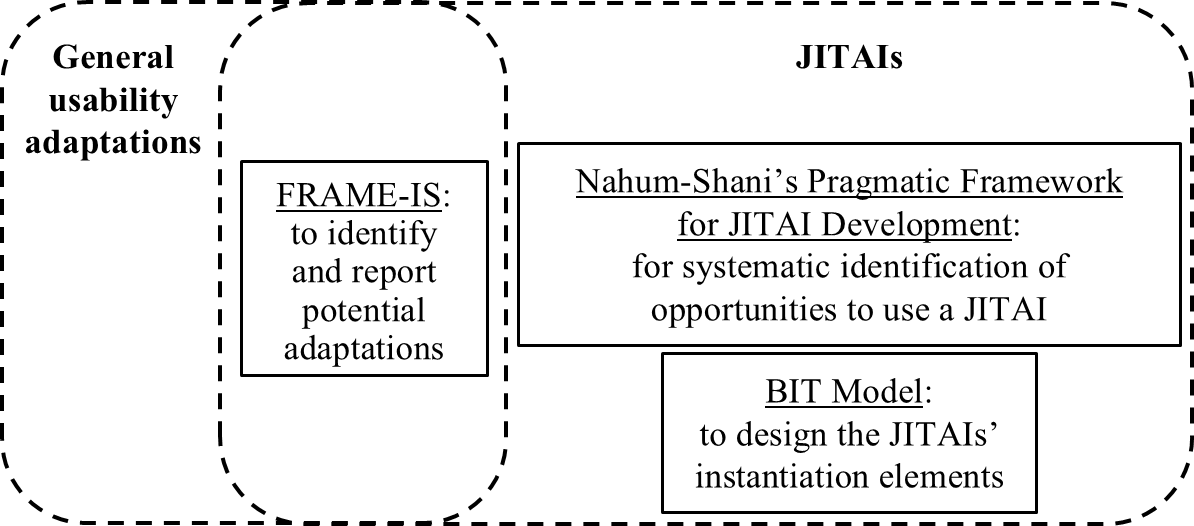
Integration of established frameworks to guide identification and development of usability-related adaptations. FRAME-IS: Framework for Reporting Adaptations and Modifications to Evidence-based Implementation Strategies; JITAI: just-in-time adaptive intervention; BIT: Behavioral Integration Technology

The Framework for Reporting Adaptations and Modifications to Evidence-based Implementation Strategies (FRAME-IS) was used to identify and report potential usability-related adaptations needed for our target user population (Table 1) (Figure 2) [49]. FRAME-IS builds from the Framework for Reporting Adaptations and Modifications-Enhanced and is organized into modules to systematically document modifications to implementation strategies [50]. We elected to use the FRAME-IS rather than the Framework for Reporting Adaptations and Modifications-Enhanced because our goal was to adapt the delivery of therapeutic content within the digital health intervention (ie, usability and engagement optimizations as an implementation strategy), rather than adapt the core therapeutic content itself (ie, cognitive behavioral therapy, mindfulness exercises, etc as the evidence-based intervention).

**Table 1.** Application of the Framework for Reporting Adaptations and Modifications to Evidence-based Implementation Strategies, which guided proposed adaptations to improve usability of a mental health app for nondigitally native adults who have chronic pain.^a^

FRAME-IS^b^ module or sub-component	Application of FRAME-IS to target intervention and population
Module 1	
The intervention being implemented is:	Therapeutic tools within the Wysa for Chronic Pain digital platform
The implementation strategy being modified is:	Improving usability of the intervention’s platform
The modifications being made are:	General adaptations to the intervention’s interface Incorporation of JITAIs^c^
The reasons for the modifications are:	To lower the user’s navigation burden and frustration through the intervention’s interface To increase the likelihood that the user is aware of, and engages with, relevant therapeutic tools
Module 2	
What is modified?	Format-related contextual modifications (See Tables 2 and 3)
Module 3	
What is the nature of the content, evaluation, or training modification?	Refining packaging of intervention content
Optional: what is the relationship to core elements?	Fidelity-consistent: core elements of the intervention are preserved
Module 4	
What are the goals?	Increase the usability and acceptability of the intervention to the target user group Improve intervention fidelity and engagement such that users engage with therapeutic tools as intended (ie, improve retention) Decrease disparities in intervention delivery (ie, improve uptake and retention by nondigitally native adults)
What is the level of the rationale for the modifications?	Recipient (end-user)
Module 5	
When is the modification initiated?	Scale-up phase (ie, after the intervention was developed but before it is made available to the general public)
Is the modification planned?	Planned, proactive
Module 6	
Who participates in the decision to modify?	Recipients (end-users), researchers, intervention developers
Optional: who makes the ultimate decision:	Intervention developers (ie, based on feasibility and alignment with other intervention features)
Module 7	
How widespread is the modification?	All recipients (end-users) of the intervention

a The left column outlines the elements of the FRAME-IS, and the right column outlines the application of the framework to this study’s target intervention and population..

b FRAME-IS: Framework for Reporting Adaptations and Modifications to Evidence-based Implementation Strategies

c JITAIs: just-in-time adaptive interventions

**Table 2.** General usability adaptations proposed to improve usability of a mental health app for nondigitally native adults who have chronic pain.

Name of adaptation	Description
Onboarding conversation	Include a thorough, text-based description of how users are intended to engage with the app (eg, timing, frequency, content, goals of engagement). Also explain terms used by the app (eg, “roadmap,” “journal,” etc).
Onboarding tour	Include a dynamic visual tour of how to navigate the app’s interface, including audio explanation and text captions.
Therapeutic approach	Explain the app’s therapeutic approach during the onboarding experience. For example, explain “how an app can help with pain” by differentiating pain interference from pain severity and by stating the goals of reducing pain interference and improving pain acceptance.
Search bar	Enable users to rapidly search the library of therapeutic tools based on a tool’s name, category, or other feature.
Clarify “SOS”	Appropriately label an app’s crisis tools so users do not assume that pushing a button labeled “SOS” will automatically dial 911 (but rather, will direct to creation and reference of user-reported sources of hope and “safe people/places,” list of crisis hotlines, etc)
Personalized “flare-up kit”	Create easy reference to a personalized plan for symptom flares (such as what would be developed by the user during a standard cognitive behavioral therapy session). For example, pin a “preparing for pain flares” plan near existing tool packs, which lists users’ preferred nonpharmacologic pain management strategies (eg, ice and heat modalities, distraction techniques, mental relaxation techniques, etc).
Pinned toolkit	Maintain a group of in-app therapeutic tools that are useful for acute needs (eg, pain flares) and are always easily accessible from the app home screen (eg, mindfulness exercises, gentle stretches, reminder of user-reported preferred pain management strategies in the “flare-up kit”).
Experience customization	Customize and recommend therapeutic tools based on users’ responses during onboarding and subsequent conversations within the app (eg, facilitated by generative artificial intelligence).
Other challenges to address	When prompting users to report challenges they are living with, include options for loneliness, health concerns, and finances (which impact overall well-being and should then be incorporated into customizing the user’s app experience).
Response to tools within app	Track users’ self-reported response to each therapeutic tool, at least after first use. Use this data for future experience customization.
Weekly symptom assessment	Assess symptoms weekly with a brief measure (eg, PHQ-4)^a^ [51], to facilitate (app- and user-led) pattern recognition between symptoms and other events (eg, to facilitate future app experience customization and for users to reference while journaling).
Weekly report	Relate symptom changes (eg, mood and pain) to app engagement (eg, which tools, how often), and allow users to add other external events to the report which they believe may have impacted their symptoms (eg, work, family events).
Preparing for hard days	Before closing the “weekly report” experience, ask users, “Do you expect any extra challenges this week?” If so, inquire about the timing and type of challenge. Offer to schedule a personalized tool in preparation for the challenge (eg, grounding exercise before a person with agoraphobia leaves home, meditation exercise for an anticipated activity-related pain flare, etc).
Tip of the day or week	As part of the daily check-in or weekly report, highlight a feature or tool that has not yet been explored by the user or that was rated highly but has not been used recently.
Curriculum change	After a user completes the app’s structured curriculum, ask if the user prefers to continue structured check-ins versus transition to only user-led engagement with tools.

a PHQ-4: Patient Health Questionnaire-4.

**Table 3.** Usability-related JITAIs proposed to improve usability of a mental health app for nondigitally native adults who have chronic pain.

JITAI^a^ name	Purpose	Tailoring variables	Intervention options	Proximal outcome(s)
Onboarding	Increase onboarding completion rates	If user downloaded app but did not complete onboarding within intended period	Then, send a notification that encourages the user to finish onboarding.	Completion of onboarding
Short disengaged	Motivate a user to engage	If no app engagement for short period (eg, 24 h)	Then, send a notification that directly opens to a relevant tool.	Any engagement^b^ Content engagement^c^
Long disengaged	Respect that users’ preferred or needed frequency of engagement may fluctuate based on symptom severity and life events	If no app engagement for a prolonged period (eg, 7 d)	Then, send an “FYI/opt^d^ back in”^e^ notification, and stop all notifications for a brief period (eg, 3 d). After the brief period, send a notification that directly opens to a relevant tool.	Any engagement^b^ Content engagement^c^
AM check-ins only	Personalize timing of content delivery to users’ demonstrated preferences	If no engagement with PM check-ins but continued engagement with AM check-ins	Then, send an “FYI/opt back in”^e^ notification, stop daily “PM check-in” notifications, and move the “activity tracking and mood reflection” task to the AM check-ins.	Engagement with the “activity tracking and mood reflection” task compared to a preceding time interval Change in content engagement^c^ compared to a preceding time interval
PM check-ins only	Personalize timing of content delivery to users’ demonstrated preferences	If no engagement with AM check-ins but continued engagement with PM check-ins	Then, send an “FYI/opt back in"^e^ notification, stop daily “AM check-in” notifications, and move the “activity scheduling and planning” task to the PM check-ins.	Engagement with the “activity scheduling and planning” task compared to a preceding time interval Change in content engagement^c^ compared to a preceding time interval
Sporadic engagement only	Personalize timing of content delivery to users’ demonstrated preferences	If no engagement with AM or PM check-ins but continued user-led engagement at other times	Then, send an “FYI/opt back in”^e^ notification, and stop daily “AM check-in” and “PM check-in” notifications. Instead, each day, send a single personalized notification that directly opens to a relevant tool.	Change in content engagement^c^ compared to a preceding time interval
Symptom health flare^f^	Offer increased support when increased need is demonstrated	If symptom score (eg, PHQ-4^g^ [51]) worsens compared to the preceding score and the user’s current push notification frequency is less than maximal frequency	Then, send an “FYI/opt out”^e^ notification, and increase notification frequency back to maximal frequency (eg, twice daily).	Change in content engagement^c^ compared to a preceding time interval
Accessibility	Reduce communication barriers	If user’s typo frequency interferes with effective communication with the app or if user’s typing speed is slower than a designated threshold	Then, send a notification that describes accessibility options (eg, increase font size, speech-to-text, text-to-speech) and offer to direct the user to their mobile device’s accessibility settings	Change in content engagement^c^ compared to a preceding time interval

a JITAI: just-in-time adaptive interventions

b “Any engagement” is operationalized as opening the app during the specified target engagement time interval. This is a binary outcome.

c “Content engagement” is operationalized as the total number of tools, conversations, and check-ins the user engaged with during the specified target engagement time interval. This is a continuous outcome.

d FYI: for your information.

e If the “opt back in” or “opt out” option is selected, the rest of the intervention is not delivered.

f The “symptom flare” JITAI over-rides any engagement-related JITAIs which would otherwise reduce the frequency of push notifications to the user.

g PHQ-4: Patient Health Questionnaire-4.

Specifically for developing usability-related JITAIs, we applied Nahum-Shani’s pragmatic framework for JITAI development and the Behavioral Integration Technology (BIT) model [52,53]. Nahum-Shani’s pragmatic framework facilitated the systematic identification of opportunities for intervention using a JITAI [52]. That is, the framework was used to operationalize intended temporal progression of events through the app, which led to identification of promising opportunities for intervention with a JITAI. The BIT model facilitates effective translation of behavioral interventions into digital delivery systems. Specifically, we used the BIT model to design the JITAIs’ instantiation elements with intentional incorporation of behavior change strategies, implementation science principles, and user interface design principles that appeal to our target population [54].

### Analysis

Using a framework-informed hybrid inductive-deductive content analysis approach [55], iterative versions of general usability adaptations and usability-related JITAIs were developed and refined throughout the usability testing process based on feedback from participants and mapping to the analysis frameworks. As usability tests were completed, audio recordings of the usability tests were professionally transcribed (Rev.com), cleaned, and chunked (CYG). Three members of the study team (ALC, CYG, AM) collaboratively conducted open coding of the first 5 transcripts using an inductive, data-driven approach to identify concepts emerging from the data. Through discussion and consensus, they developed an initial codebook, which was then applied independently to pilot code the next 3 transcripts. Interrater reliability for these three transcripts was approximately 90%, with fewer than 5 coding discrepancies across coders. Discrepancies were resolved through consensus, and the codebook was refined accordingly. As usability tests were conducted with additional participants, the finalized codebook was applied using an independent open coding approach to these additional transcripts, each of which was coded by a single team member (CYG or AM), with periodic cross-checks between coders to ensure coding consistency. Coding was managed using NVivo 15 (Lumivero). Three team members (ALC, CYG, and AM) collaboratively and iteratively grouped related codes into preliminary themes that reflected participants’ usability needs and suggested app adaptations, including JITAIs. This process was continued until analysis of additional transcripts no longer yielded new themes. Then, we conducted a deductive analysis by examining the inductively derived themes in relation to the FRAME-IS framework, Nahum-Shani’s pragmatic framework for JITAI development, and the BIT model. The 3 team members (ALC, CYG, and AM) reviewed and discussed the themes to determine their alignment with these guiding frameworks, refine interpretation where appropriate, and translate findings into a final set of proposed general usability adaptations and usability-related JITAIs.

### Reflexivity Statement

Usability testing was facilitated by a senior female research coordinator who has clinical research experience working with older adults and in the fields of mental health and orthopedics (AM). Participants’ only contact with the facilitator was related to study recruitment, consent, and participation. The other 2 research team members who contributed to codebook development included a female physician scientist with a clinical background in physical medicine and rehabilitation (ALC), as well as a female physician trainee in physical medicine and rehabilitation residency (CYG). The clinical and musculoskeletal background of these team members may have influenced data collection and interpretation such that physical barriers and related adaptations were disproportionately considered over solutions that considered mental health and systems-level factors. We worked to balance this potential bias by incorporating input from a clinician scientist with mental health clinical expertise (SH) and a health information technology expert (JA).

### Rigor and Reproducibility

Informal member checking was performed by the facilitator (AM), who paraphrased and restated participants’ feedback during the usability testing process to confirm that participants’ intent and insights were accurately understood. Additionally, preliminary themes that emerged from early interviews were shared with later participants to assess for perceived validity. Participants generally affirmed the face validity of these themes.

## Results

Of the 62 people who completed the screening questionnaire and were determined to be eligible, 42 users participated in usability testing (mean age 57, SD 8 years; women: n=32, 76%; mean chronic pain duration 16, SD 12 years) (Table 4). Reasons for nonparticipation included (1) not responding to invitations to schedule an appointment (or missing a scheduled appointment) (n=4), and (2) achievement of thematic saturation prior to their participation (n=16).

**Table 4.** Characteristics of usability testing participants (N=42).

Characteristics	Values
Age (years), median (IQR; range)	55 (51-61; 45-76)
Sex^a^, n (%)	
Female	32 (76)
Male	10 (24)
Race, n (%)	
White	30 (71)
Black or African American	9 (21)
Other	1 (2)
More than one race	1 (2)
Unknown or prefer not to answer	1 (2)
Ethnicity, n (%)	
Hispanic or Latino	3 (7)
Not Hispanic or Latino	36 (86)
Unknown or prefer not to answer	3 (7)
Employment status, n (%)	
Working full-time	11 (26)
Working part-time	4 (10)
Disabled	13 (31)
Retired	10 (24)
Unemployed	2 (5)
Prefer not to answer	2 (5)
Frequency of internet use^b^, n (%)	
Most of the day	12 (29)
Multiple times a day	28 (67)
At least once a day	1 (2)
Less than once a day	1 (2)
High-impact chronic pain present^c^, n (%)	33 (80)
BPI Pain Interference score^d^, median (IQR; range)	7.1 (6‐8.1; 2.4‐9)
PHQ-9^e^ score (years), median (IQR; range)	14 (12-17; 7-22)
GAD-7^f^ score (years), median (IQR; range)	12 (9-15; 1-21)

a Sex and gender identity were concordant for all participants.

b Self-reported internet use was captured by the Pew Digital Savviness Classifier [56].

c ”High impact chronic pain” is defined as pain that limits life or work activities on most or every day over the past 3 months [20].

d The Brief Pain Inventory (BPI) Pain Interference score ranges from 0 to 10 [57]. Higher scores indicate greater pain interference.

e The Patient Health Questionnaire (PHQ)-9 is a 9-item screening measure for depressive symptoms [39]. Scores range from 0 to 27. Higher scores suggest more depressive symptoms.

f The Generalized Anxiety Disorder (GAD)-7 is a 7-item screening measure for anxiety symptoms [40]. Scores range from 0 to 21. Higher scores suggest more anxiety symptoms.

We identified general usability adaptations, as well as usability-related JITAIs, that users perceived would enhance their app experience. Rapid, nearly universal convergence was achieved related to suggestions for general adaptations to provide a more thorough onboarding experience to the app (Table 2). Specifically, users requested clear, step-by-step navigation instructions, rather than a brief onboarding experience that relied on user-led exploration of the app and familiarity with conventional app symbols (eg, three vertical ellipsis dots indicating “more options”). A 49-year-old female stated:

For those who may not be as familiar – and you’re going to start getting a lot of those much older users, especially in this kind of a world with health concerns – I don’t know that they would know what that [icon] means without a tour of the app first.

Users also appreciated a customized app experience based on their unique challenges and app usage patterns, although specific desired customizations were somewhat varied, and no clear patterns emerged related to demographic characteristics or recruitment source, based on impressionistic observation.

Consistent with Nahum-Shani’s pragmatic framework [52], the majority of participants favored that some of the usability-related JITAI opportunities be designed to strategically reduce notifications to the user in order to reduce the likelihood of a negative impact such as notification fatigue and subsequent complete disengagement with the app (Table 5). As one 60-year-old female participant said:

If I’ve not engaged in a week and after three days of nothing bothering me – no notifications – I think that a notification out of the blue would probably kick me back into gear. Give me that little kick start I need.

When a notification is delivered, application of the BIT model [53] facilitated identification of the notification’s instantiation components that were most appealing to users (Table 6).

**Table 5.** Application of Nahum-Shani’s pragmatic framework which guided development of usability-related JITAIs to improve usability of a mental health app for nondigitally native adults who have chronic pain.

Component of Nahum-Shani’s pragmatic framework	Application to target intervention and population
Defining the problem	
Whom are you trying to help?	Nondigitally native adults, especially those who have chronic pain that coexists with symptoms of depression, anxiety, or both. This target population may be less likely than younger adults to readily and proficiently navigate through an app independently and find tools of interest.
What is the desired distal outcome of the JITAI(^c^s)?	Improve depression and anxiety symptoms (primary outcomes) Improve behavioral activation, pain acceptance, and sleep quality (proposed mediators of primary outcomes)
What is the temporal progression of key factors towards the distal outcome?^b^	Morning engagement: for behavioral activation (eg, activity scheduling and planning) Evening engagement: for behavioral activation (eg, activity tracking, mood reflection), sleep optimization (eg, meditation) Weekly engagement: for reflection and introduction of new cognitive behavioral skills (eg, cognitive reframing, thought recording) Sustained engagement (8 wk): for growth, maintenance, and eventual independence with cognitive behavioral and pain self-management skills
What are contender proximal outcomes?	Tool-specific engagement within the app
Defining just-in-time in the context of the identified problem	
What factors mark a state of vulnerability or opportunity?	Lack of engagement with the app (as intended or as previously engaged)
What possible intervention options can affect the proximal outcomes?	A notification or nudge that is noticed by, and appeals to, the user
What factors mark a state of unreceptivity to the selected intervention options?	Already engaging with the app as intended or more frequently Not responding to existing notifications or nudges
Formulating the adaptation strategy:	
What are the tailoring variables?	User’s current pattern of engagement
For each possible level of the tailoring variable, which intervention option is likely to have the desired effect on the proximal outcome?	See Table 3. Additional research is needed to identify optimal cut-points for each tailoring variable.
What plausible decision rules can be generated to operationalize effective adaptation?	See Table 3. Additional research is needed to identify optimal cut-points for each tailoring variable.

a JITAI: just-in-time adaptive interventions.

b The temporal progression described reflects how users are *intended* to engage with the app.

**Table 6.** Application of the BIT^a^ model which guided development of usability-related JITAIs^b^ to improve usability of a mental health app for nondigitally native adults who have chronic pain.

BIT component	Application to target intervention and population
Theoretical	
Why: aims	
Clinical aims	Improve depression and anxiety symptoms
Usage aims	Increase engagement with evidence-based therapeutic tools
How (conceptual): behavior change strategies	
Clinical strategies	Improve behavioral activation (eg, through goal setting), pain acceptance (eg, through pain neuroscience education and mindfulness exercises), and sleep quality (eg, through sleep hygiene education and meditation exercises)
Usage strategies	Direct user through a structured educational curriculum and on-demand access to therapeutic tools
Instantiation	
What: elements	Notifications that are delivered with personalized timing and content based on passively and actively collected data
How (technical): characteristics	Each push notification will feature a visual and audio alert, as permitted by the user’s self-selected mobile device settings. The notifications direct the user to relevant therapeutic tools, as identified from the user’s prior responses (eg, challenges expressed) and app engagement patterns. The content of the notifications is encouraging but not overly cheerful, with judicious use of emojis.
When: workflow	Timing of delivery of the push notifications is determined by event-based rules. However, precise timing relative to occurrence of the event(s) may be adaptive based on the user’s engagement history and other characteristics.

a BIT: behavioral intervention technology

b JITAI: just-in-time adaptive intervention.

We identified 8 potential usability-related JITAIs to consider for build-out and formal effectiveness testing (Table 3). These JITAIs were developed to address the competing priorities of (1) providing predictable support to the user, and (2) acknowledging that pain and mood symptoms (and hence, a user’s need for support) can fluctuate. Users reported varying preferences regarding the frequency of push notifications and indications to change the push notification frequency. Therefore, our JITAIs that propose to change the frequency of future push notifications all include an “opt back in” or “opt out” option so that users maintain autonomy over their app experience. As one 48-year-old male described:

Some people, if they’re in a certain mental state, may need a little bit more persistence, whereas other people may need more space. I feel like me personally – if I didn’t engage for a while and then had a [several-day break from notifications], it would be like, ‘Hey, we’re still here if you need us.’ I think that would work for me personally, but I think it would all depend on the individual. If the app gave you an option or the ability to change the [notification frequency] or suggest how you want to be taken care of, yeah, I think that would be a good idea.

The precise text of each JITAI notification is intended to be engaging and varied over time, even if the same JITAI is triggered more than once. As relevant and possible, the JITAI notifications should leverage information that was previously reported by the user in order to personalize the content and resonate with the user (eg, suggesting tools based on prior challenges reported, tools that were previously rated as helpful, and tools that have not been used yet but are expected to be helpful based on the user’s reported challenges and prior tools used). When selected, most of the JITAI notifications are intended to directly open the app to a relevant tool for the user.

## Discussion

### Principal Results

In this study, we identified adaptations to an existing mental health app that better meet the stated usability preferences and needs of nondigitally native adults who have chronic pain and coexisting symptoms of depression or anxiety. Study participants specifically identified several opportunities to optimize their user experience by ensuring that the digital health intervention clearly describes how all its features are intended to be used and by minimizing navigation burden. For instance, users proposed general usability adaptations for a more robust orientation experience during app onboarding and for ongoing personalized engagement prompts using usability-related JITAIs.

### Comparison With Prior Work

Based on prior literature and feedback from participants in this study [15,58], when identifying decision rules for our proposed JITAIs, we aimed to balance the use of passively collected app engagement data and user-directed triggers in order to respect users’ dual desires for reduced navigation burden and also maintenance of autonomy and privacy. In related work [12], the majority of users in this target population expressed hesitation about allowing a digital mental health app to collect and use other data, such as physical location that is passively collected from their mobile device or other wearable sensors. Furthermore, older adults and those with multimorbidity are less likely to continuously carry a mobile device or use a wearable sensor, which would limit the impact of JITAIs that rely on datapoints from these sources [59-62]. As a result, the JITAIs developed in this study were intentionally designed to operate using only engagement-related and user-reported data that is collected within the app itself.

From a practical standpoint, the proposed JITAIs identified in this study represent general concepts that were perceived favorably by users. Determining the effectiveness of the proposed JITAIs and identifying ideal specific cut-points for each JITAI’s tailoring variables (eg, number of hours or days of no app engagement before delivering an engagement-related JITAI) requires additional investigation. Although preliminary cutoff points for proposed JITAIs can be developed based on qualitative evidence from a usability study such as this, recommended best practices suggest follow-up investigation using complex analytical techniques to refine evidence-based (and potentially adaptive) cutoff points [15]. Implementation of potential JITAIs also depends on feasibility within the digital health platform, as well as confirmation that the final decision rules and cutoff points of the JITAIs do not conflict with one another or with other elements of the intervention’s features or user experience. We plan to refine the details of these JITAIs using a series of microrandomized trials and additional qualitative user feedback [41].

In addition to leveraging existing evidence-based frameworks, we also leveraged an academic-industry partnership to expedite the incorporation of evidence-based principles into a digital health intervention with potential for rapid dissemination to the general public [63]. Specifically, the commercial app company facilitated participant access to the intervention and iterative mock-ups for the usability testing. The academic researchers led the usability testing itself and applied evidence-based frameworks to guide data collection and analysis. After the identified adaptations are fully built and subsequently tested for effectiveness, they can rapidly be rolled out to several thousand daily users in a much faster time scale than would be possible if an academic group developed a digital health intervention de novo. Our process to identify implementable usability-related adaptations of an existing mental health app can serve as a proof-of-concept method for relatively rapid refinement of other existing digital health interventions in order to better serve specific target populations, especially for apps that already have robust evidence-based content but could benefit from adaptations for specific subpopulations.

A major strength of this study is the integrated framework-based approach to identify usability-related opportunities for a target user population with unique needs. This approach can serve as a model for other studies, and the usability-related principles that were identified in this study are broadly generalizable for other digital health interventions that are intended for nondigitally native adults with multimorbidity. The robust sample size is also a strength relative to other digital health usability studies [64,65].

### Limitations

Some limitations are inherent to the study design used. Observer bias (ie, the Hawthorne effect) may have resulted in participants reporting more confidence or demonstrating greater tenacity with technical features than they otherwise would in a real-world setting. Additionally, the iterative nature of our usability testing resulted in some heterogeneity in participant experiences. While participants were not exposed to identical interfaces, this approach is consistent with user-centered and formative usability evaluation methods, in which iterative refinement is prioritized to rapidly identify and address usability barriers [66]. Furthermore, details of some of the usability-related adaptations and JITAIs that were identified in this study are predominantly applicable to the specific mental health app that was tested. Our proposed adaptations also reflect participants’ stated preferences, but quantitative assessment and data-driven refinement was outside the scope of this study. Finally, user preferences among nondigitally native adults may evolve over time as the population’s overall experience with technology continues to grow.

Given the demographics of our sample, our study findings are likely most generalizable to middle-aged adults in the United States who are relatively digitally proficient and already use the internet routinely throughout the day. As a result, findings from this study may not fully capture the usability needs and other barriers faced by older adults with low digital literacy, which remains an important and underserved segment of the target user population. Interpretation of generalizability is also limited by the self-selected nature of the convenience sample and the lack of a standardized cognitive screening instrument, especially since the prevalence of mild cognitive impairment increases with age.

### Conclusions

Identifying implementable opportunities to improve usability and prompt meaningful engagement at relevant timepoints holds promise to improve the effectiveness of digital health interventions because intervention attrition is a well-known challenge that is suspected to reduce the effectiveness of digital health interventions on a population level [67-70]. In this study, we used an integrated framework-based approach and user feedback to identify usability-related optimizations to an existing mental health app that are preferred among relatively digitally proficient but nondigitally native adults who have chronic pain and coexisting symptoms of depression or anxiety. Users identified general usability-related adaptations and usability-related JITAIs which were appealing for their potential to (1) provide clarity with regard to how to navigate the digital health intervention, and (2) reduce the user’s burden of independent navigation within the intervention. These usability-related refinement opportunities are generalizable to other digital health interventions intended for older users who are likely to be managing multimorbidity, are not digital natives, and are more likely to have physical or cognitive impairments than younger users (eg, medication reminder apps, daily safety monitoring apps, etc). The integrated framework-based approach taken in this study can also serve as a model for refinement of digital health interventions with other target populations, as well.

## Supplementary material

10.2196/87365 Multimedia Appendix 1 Interview guide.

## Abbreviations:

BIT: behavioral integration technology
FRAME-IS: Framework for Reporting Adaptations and Modifications to Evidence-based Implementation Strategies
JITAI: just-in-time adaptive intervention
REDCap: Research Electronic Database Capture
