# Supplementary material for: Optimizing Usability of Digital Health Interventions for Nondigitally Native Adults: A Framework-Based Approach to Develop Just-in-Time Adaptive Interventions (JITAIs) and Other Adaptations
Source: JMIR Hum Factors. 2026 Aug 11;13:e87365. doi: 10.2196/87365 (PMC13460799; doi:10.2196/87365)
Supplement: Supplementary file 1 [file humanfactors-v13-e87365-s001.docx]

**Optimizing usability of digital health interventions for middle-aged and older adults: A framework-based approach to develop usability-related adaptations including just-in-time adaptive interventions (JITAIs)**

**INTERVIEW GUIDE FOR USABILITY TESTING**

*[Tasks]*

1. Understanding navigation:
   a. Please download the app and complete the onboarding process.
   b. Now that you’ve finished onboarding to the app, you’ll be taken to this “home screen.” What would you do with the app once you get to this screen?
   c. Please explore the app and share your thoughts as you do so.
   d. If you wanted to change any selections that you made during onboarding, how would you do that?

2. Tool packs (and chatbot conversation):
   a. Next, I’d like you to explore the tool packs. Choose one that you’d like to try, and then engage with it. Please share your thoughts as you do so.
   b. What other tool packs or features would you look for in an app like this?
3. Check-in:
   a. This is a check-in notification and screen. How often would you want to receive a message like this?
   b. Please share your thoughts as you follow the instructions.
   c. This app is meant to be used at least 3 times weekly, but it encourages users to engage briefly every morning and evening. How often would you want reminders from the app to use it? What circumstances might affect how often you want reminders from the app, and how might you want the app to respond?

4. Roadmap:
   a. What is your understanding of what this “Roadmap” screen is showing you?
   b. What does (or would) make this roadmap meaningful/helpful/clear/motivating to you?
5. Report:
   a. What is your understanding of this “Report” notification and screen?
   b. What sort of information would you want to see on a progress report?
   c. How often would you want to receive a progress report like this?
6. SOS:
   a. If you had strong thoughts of hurting yourself or otherwise felt you were in a mental health crisis, can you show me what you might do in the app?

*[JITAIs]*

7. Dis-engagement: Right now, this app sends notifications twice a day to remind you to interact with it. Depending on a person’s circumstances, stress and pain levels, busyness, and preferences, we imagine there may be times where this twice daily frequency is helpful and other times when it isn’t. Imagine you haven’t used the app at all for a while. What would you think if those notifications stopped for a while. Then at some time later, you get a customized notification that offers support for one of the challenges that you described when you onboarded to the app.
   a. How does that sound to you?
   b. How do you think it might affect whether you use the app?
8. Mental health symptom severity: Right now, this app sends notifications twice a day to remind you to interact with it, no matter how you’re feeling. Imagine that the app checked in with you more often when you’re feeling worse and less often when you’re feeling better.
   a. How does that sound to you?
   b. How do you think it might affect whether you use the app?
   c. How would you prefer the app “know” when you’re feeling worse?
   d. If it helped the app be more useful for you, what kind of information would you feel comfortable with the app collecting about you based on how you use your mobile device (after asking for permission)?
9. Accessibility: Suppose you have difficulty pushing the buttons or reading the words on your phone, for instance due to hand pain or stiffness, or vision trouble. If the app notices that you’re typing slowly, responding slowly, or having a lot of typos, imagine that the app gave a suggestion for something like, “Would you like to make my words (or the letter keys) bigger?” or “Would you like me to say this conversation out loud, rather than write it on the screen?” or “Do you want to try a darker screen?”
   a. How does that sound to you?
   b. How do you think it might affect whether you use the app?
10. Sleep: Imagine that when you downloaded this app, you reported that sleep was a challenge for you. The app has a feature called a “sleep routine” to help users fall asleep. It can be used every night, but let’s suppose you haven’t used it yet, or you’re using your mobile device past when you usually stop using it at night. What would you think if you received a message from the app that said, “Hi! It’s getting late. Would you like to wind down with a sleep routine?”
    a. How does that sound to you?
    b. How do you think it might affect whether you use the app?
11. Hard days: Sometimes we know something challenging is coming up, even though it hasn’t happened yet – like a medical procedure, an activity that will flare pain, or an unwanted social gathering or hard conversation. Imagine that the app asked you something like, “Does today feel like an extra hard day?” or “Are you facing extra challenges today?” And if you answer, “Yes,” the app asks what kind of extra challenges you’re facing– like extra pain, anxiety, depressed mood, or something else. Based on your answer, the app can offer a tool that is especially designed to help with the kind of challenge you’re dealing with. For example, deep breathing for pain, mindfulness for anxiety, or scheduling an activity that brings joy for depressed mood. You could choose to do the tool at that time, or you could schedule it for later when you expect that challenge to come.
    a. How does that sound to you?
    b. How do you think it might affect whether you use the app?
12. Tip/tool of the week: Imagine that each week, you got one notification that was personalized to the challenges you described when you first downloaded the app. For instance, if you said that panic was a challenge for you but you had not yet used the “Grounding for Panic” tool, you might get a notification like, “Tip of the week: Feeling panicked? Try ‘Grounding for Panic’ to help calm your mind and body.”
    a. How does that sound to you?
    b. How do you think it might affect whether you use the app?
